# Persistent depressive symptoms, HPA-axis hyperactivity, and inflammation: the role of cognitive-affective and somatic symptoms

**DOI:** 10.1038/s41380-019-0501-6

**Published:** 2019-08-21

**Authors:** Eleonora Iob, Clemens Kirschbaum, Andrew Steptoe

**Affiliations:** 1 Department of Behavioural Science and Health, University College London, London, UK; 2 Department of Psychology, Technische Universität Dresden, Dresden, Germany

**Keywords:** Psychology, Predictive markers

## Abstract

Hypothalamic-pituitary-adrenal (HPA)-axis hyperactivity and inflammation are thought to be prominent in the aetiology of depression. Although meta-analyses have confirmed this relationship, there is considerable variability in the effect sizes across studies. This could be attributed to a differential role of such biological systems in somatic versus cognitive-affective depressive symptoms which remains largely unexplored. Furthermore, most longitudinal research to date has focused on transient rather than persistent depressive symptoms. In the current study, we investigated the associations of hair cortisol and plasma C-reactive protein (CRP) with the longitudinal persistence and dimensions (cognitive-affective versus somatic) of depressive symptoms over a 14-year period using Trait‐State‐Occasion (TSO) structural equation modelling. The data came from a large sample of older adults from the English Longitudinal Study of Ageing. Depressive symptoms were assessed from wave 1 (2002–03) to wave 8 (2016–17). Hair cortisol (*N* = 4761) and plasma CRP (*N* = 5784) were measured in wave 6 (2012–13). Covariates included demographic, socioeconomic, lifestyle, chronic disease, and medication data. Our results revealed that higher cortisol and CRP levels were significantly associated with persistent depressive symptoms across the study period. Notably, both biomarkers exhibited stronger relationships with somatic than with cognitive-affective symptoms. The associations with somatic symptoms were also independent of relevant confounding factors. In contrast, their associations with cognitive-affective symptoms were weak after adjustment for all covariates. These distinct associations reveal the importance of considering symptom-specific effects in future studies on pathophysiological mechanisms. Ultimately, this will have the potential to advance the search for biomarkers of depression and facilitate more targeted treatments.

## Introduction

Depression is a common mental disorder characterised by an array of cognitive, affective, and somatic symptoms [1, 2]. Exposure to stressful life circumstances is one of the strongest risk factors for the development of depression. For instance, it has been demonstrated that high levels of psychosocial stress can affect not only the first onset of depression, but also its severity, remission, or relapse [3, 4]. Besides this, several meta-analyses have demonstrated that depression has a bidirectional relationship with numerous chronic disease outcomes [5–7]. The relationship of depression with stress and physical health is particularly relevant at older ages due to increasing stressors resulting from declining physical health and diminishing social connections [8, 9]. Hence, with a progressively ageing population worldwide, understanding the biological mechanisms underlying the links between stress, depression, and physical health at older ages becomes increasingly important [10].

Biological systems that are thought to be prominent in the aetiology of depression and underlie its relationship with stress and physical health include the hypothalamic-pituitary-adrenal (HPA)-axis and the inflammatory response system [11]. Activation of the HPA-axis is a hallmark of the stress response in humans [12], representing an important indicator of psychosocial stress [13]. Convincing evidence also suggests that exposure to stress can result in elevated inflammatory responses in the brain as well as peripherally [14, 15]. Accordingly, an abundance of studies has indicated that depressed individuals tend to exhibit elevated cortisol levels [16] and greater plasma concentrations of pro-inflammatory cytokines such as C-reactive protein (CRP) [17]. In addition to their associations with stress and depression, HPA-axis hyperactivity and elevated inflammation have also been implicated in the development of various cardiometabolic, inflammatory, endocrine, and neural disorders [18–21].

Although meta-analytic studies have confirmed the association of depression with HPA-axis hyperactivity and inflammation, there is considerable variability in the effect sizes across studies, which overall appear to be weaker than is often assumed [16, 22, 23]. This could be attributed to differences in the study samples (e.g., clinical versus population based), or to methodological differences in the measurement of depression [24]. Another possibility is that most studies have focused on depression as a whole thereby neglecting possible symptom-specific associations [25]. Depression is in fact a highly heterogeneous disorder in terms of varying and sometimes opposing symptoms [26]. Different types of cognitive-affective and somatic symptoms are outlined both in the ICD-10^2^ and DSM-5^1^ diagnostic criteria for depression. Similarly, factor analytic studies of various depression scales have found evidence for distinct somatic and cognitive-affective components although in different combinations [27]. Despite this, individual differences in depressive symptom profiles have typically been ignored owing to the prevailing use of sum scores [25].

According to the sickness behaviour theory [28, 29], upregulated inflammatory and neuroendocrine responses can result in somatic depressive-like symptoms ﻿such as fatigue, sleep problems, lack of appetite, and psychomotor slowing, which are often referred to as ‘sickness behaviour’ in the experimental animal literature [30]. Accordingly, there is some preliminary evidence suggesting that the relationship of depression with HPA-axis hyperactivity and inflammation might be predominantly driven by somatic symptoms (e.g., tiredness, lack of energy, and sleep problems), whilst associations with cognitive-affective symptoms (e.g., sadness, positive affect, and depressed mood) are considerably lower [31–35]. Thus, elevated inflammatory and neuroendocrine responses might contribute to the pathogenesis of the somatic components of depression [32, 34]. Another important limitation is that most studies have used cross-sectional assessments of depression which cannot disentangle persistent from episodic depressive symptoms. In fact, there is substantial variability in the duration and chronicity of symptoms amongst people with identical diagnoses, as well as amongst those who do not meet diagnostic criteria. For example, some individuals may experience depressive symptoms occasionally, whereas others may exhibit persistently high levels of symptoms [36, 37]. Importantly, persistent depressive symptoms are likely to be a more robust indicator of the accumulation of biological risk factors influencing depression [37, 38].

Therefore, the aim of the present study was to investigate the associations of hair cortisol and plasma CRP with depressive symptoms over a 14-year period in a large representative population cohort of older adults. For this purpose, we used Trait‐State‐Occasion (TSO) structural equation modelling [39], which allowed us to measure the persistence, as well as dimensions (i.e., cognitive-affective and somatic) of depressive symptoms over time. We hypothesised that greater persistence of overall depressive symptoms would be associated with higher cortisol and CRP levels. Furthermore, we expected that both cortisol and CRP would exhibit stronger effects on somatic than on cognitive-affective symptoms.

## Materials and methods

### Sample

The English Longitudinal Study of Ageing (ELSA) is an ongoing, multidisciplinary prospective cohort study of women and men aged 50 years and over living in England [40]. A description of the data collection methods and sample design can be found at www.elsa-project.ac.uk. Depressive symptoms were assessed from wave 1 (2002–03) to wave 8 (2016–17), while hair cortisol and plasma CRP measurements were made during the nurse visit in wave 6 (2012–13). ﻿Out of the 7699 participants who participated in the nurse interview, hair samples were collected from 5451 individuals. However, 690 cases were excluded since they had undetectable or extreme (>660 pg/ml) cortisol values, resulting in a sample of 4761 participants. Blood samples for the measurement of CRP were collected from 6126 participants. Study members with CRP values > 10 mg/L were excluded from the analysis since this may reflect immune activation due to current infection rather than chronic inflammation. Thus, the final CRP sample included 5784 participants. All respondents provided informed consent and ethical approval was obtained from the National Research Ethics Service [41]. The ELSA datasets can be accessed through the UK Data Service (www.ukdataservice.ac.uk).

### Depressive symptoms

Depressive symptoms were ascertained using the 8-item Centre for Epidemiological Studies-Depression scale (CESD-8) [42]. CESD-8 scores equal or greater than three correspond with the traditional CESD-20 cut-off of 16 points for a clinical diagnosis of depression [43, 44]. At each wave, we calculated a cognitive-affective score (“enjoyed life”, “felt depressed”, “happy”, “lonely”, and “felt sad”) and a somatic score (“everything I did was an effort”, “sleep was restless”, and “I could not get going”) representing the total number of depressive symptoms reported by the participant for each dimension. To further validate this two-dimensional model found in previous exploratory analyses [27], we performed a confirmatory factor analysis (CFA) of the CESD-8 items using the full ELSA sample at wave 6 (*N* = 8031).

### Biological measures

#### Hair cortisol

Hair strands of ∼3 cm and weighing at least 10 mg were collected from the posterior vertex as close to the scalp as possible. Exclusion criteria for hair sampling included: ﻿pregnancy, breastfeeding, certain scalp conditions, having <2 cm of hair length, and inability to sit with head remaining still. Assuming an average hair growth of ∼1 cm per month [45], the 3 cm hair segment closest to the scalp provides a measure of the average cortisol output over the 3 months prior to sampling. The hair analysis was conducted by the Technische Universität Dresden (Germany) in two separate phases (2015 and 2018) due to financial constraints. Cortisol levels were quantified by high performance liquid chromatography–mass spectrometry following a standard wash and steroid extraction procedure [46], and were expressed in pg/mg.

#### Plasma CRP

High sensitivity plasma CRP was assayed using the N Latex CRP mono immunoassay on the Behring Nephelometer II Analyzer (Dade Behring, Milton Keynes, UK) [47]. Exclusion criteria for blood sampling included: clotting or bleeding disorders, history of fits or convulsions, or being on anticoagulant medication [48]. For the purpose of this analysis, CRP concentration was expressed in mg/L.

### Covariates

The analyses were adjusted for relevant demographic, socioeconomic, lifestyle, health, and medication data measured in wave 6. These included: sex, age, wealth, smoking status, physical activity, frequency of alcohol use, body mass index, presence of chronic diseases (i.e., cardiovascular conditions, cancers, chronic lung disease, and diabetes), use of anti-inflammatory or antihypertensive drugs, and antidepressants. The models including hair cortisol were also adjusted for hair-related characteristics (i.e., whether hair was dyed, season of hair collection, and phase of hair analysis).

### Statistical analyses

**Fig. 1:**
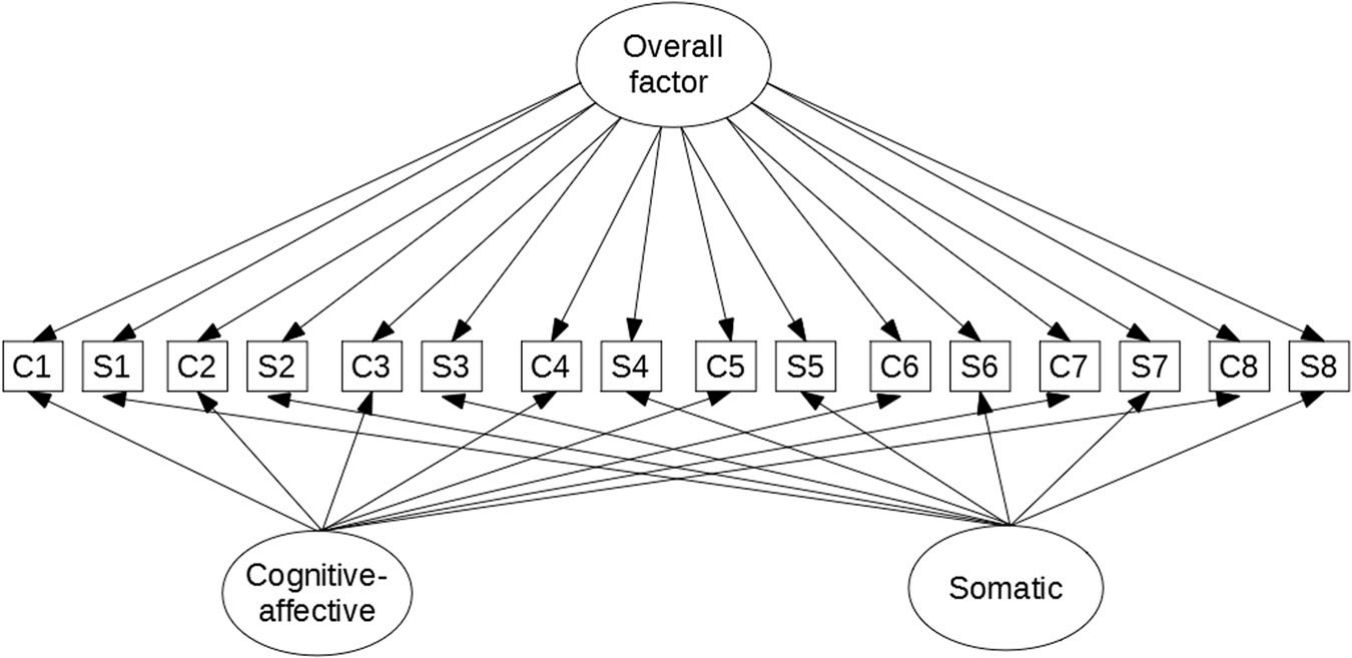
Trait-State-Occasion (TSO) model of depressive symptoms. Simplified illustration, eFigure 1 in SI file for full specification model. *C* = cognitive-affective score. *S* = somatic score. Squares represent observed variables; circles represent latent factors. The overall factor measures the longitudinal persistence of depressive symptoms. The cognitive-affective and somatic factors correspond to the two symptom-specific dimensions

The cortisol and CRP measures were log transformed since their distribution was positively skewed. All continuous variables were standardised and mean centred. The longitudinal persistence and dimensions of depressive symptoms were measured using TSO structural equation modelling [39, 49] based on the observed cognitive-affective and somatic scores at each wave (Fig. 1). The complete specification model used in the analysis is shown in eFigure 1 [Supplementary Information (SI)] as described in Newsom (2015) [49]. Since the observed scores were treated as ordinal variables, the models were fitted using the robust weighted least squares estimator, which handles missing data by estimating parameters and standard errors directly from the available data under the MARX assumption (i.e., missing at random with respect to the covariate variables) [50]. In a second step, the three latent factors representing overall, cognitive-affective, and somatic depressive symptoms were used as outcomes in a full structural equation model to test their associations with cortisol and CRP controlling for relevant confounders. The effects of cortisol and CRP on the latent factors were analysed in separate models. Data management was conducted in Rstudio version 3.4.4. The TSO models were estimated using Mplus version 7. Further details about the statistical analyses, model fit indices, and coding of the covariates can be found in the SI file.

## Results

**Fig. 2:**
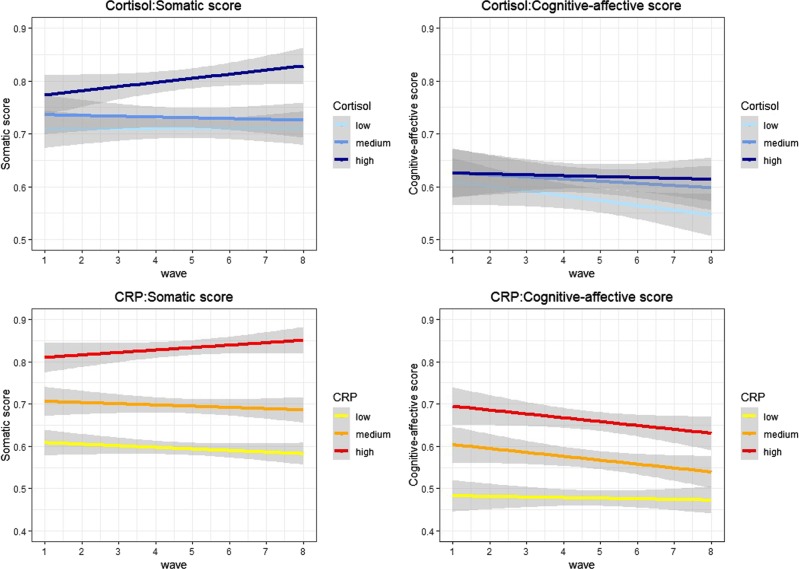
Mean scores of somatic and cognitive-affective depressive symptoms at each wave (1–8) by hair cortisol and CRP tertiles. Data source: ELSA, waves 1–8. CRP = C-reactive protein. The trajectories of the mean scores were estimated using a smoothing function with linear regression. The grey bands represent the confidence intervals of the trajectories. The data presented in this graph are for descriptive purposes only and do not relate to the trait-state-occasion models tested in the main analysis

Descriptive statistics of the study participants are provided in Table 1. The average age was 67 years in the cortisol sample (67% female) and 66 years in the CRP sample (55% female). There was a higher proportion of participants in the highest compared with the lowest wealth quintiles. Depressive symptoms were generally low, but ranged across the full spectrum from zero to eight. The percentage of participants with high depressive symptoms (mean total CESD-8 score ≥ 3) across waves 1–8 was 16% in the cortisol sample and 14% in the CRP sample. Figure 2 displays the mean somatic and cognitive-affective scores at each wave of data collection by cortisol and CRP tertiles for illustrative purposes only.

**Table 1:** Sample characteristics

		Cortisol sample (*N* = 4761)			C-reactive protein sample (*N* = 5784)		
Variables	Levels	Missing (%)	Mean (sd)	Frequency (%)	Missing (%)	Mean (sd)	Frequency (%)
Depressive symptoms (CESD-8)							
Overall score		1.0	1.20 (1.69)		0.8	1.13 (1.64)	
Cognitive-affective score		1.0	0.60 (1.19)		0.7	0.57 (1.15)	
Somatic score		0.4	0.71 (0.94)		0.3	0.67 (0.91)	
Stress biomarkers							
Hair cortisol (log, pg/mg)		–	0.89 (0.56)		39.5	0.88 (0.57)	
Plasma C-reactive protein (log, <10 mg/L)		26.6	0.37 (0.92)		–	0.36 (0.92)	
Demographics							
Sex	Men	–		33.0			45.4
	Women			67.0			54.7
Age		–	67.47 (9.39)		–	66.41 (9.09)	
Wealth (quintiles)	1 (lowest)	1.7		18.0	1.7		17.1
	2			19.6			19.4
	3			20.3			20.7
	4			21.0			21.5
	5 (highest)			21.2			21.3
Lifestyle indicators							
Current smoker		–		11.0	–		11.1
Physical activity	Low	–		60.2	–		56.1
	High			39.8			43.9
Alcohol use (frequency)		8.3	4.44 (2.23)		8.1	4.26 (2.17)	
Body mass index (BMI)		4.3	28.27 (5.40)		2.9	27.98 (4.95)	
Chronic conditions							
CVD		–		22.6	–		18.8
Cancer		–		5.4	–		4.6
Chronic lung disease		–		4.4	–		4.1
Diabetes		–		10.3	–		9.2
Medications							
Anti-inflammatory/antihypertensive		–		45.3	–		42.9
Antidepressants		–		11.8	–		10.5
Hair characteristics							
Hair dyed		0.6		33.6	–		–
Season hair collection	Summer	–		23.4	–		–
	Autumn			42.7			
	Winter			26.7			
	Spring			7.2			
Phase of hair analysis	1 (2015)	–		53.6	–		–
	2 (2018)			46.4			

Data source: ELSA, wave 6. *sd* standard deviation, *CESD*-8 eight-item centre for epidemiological studies-depression scale, *CVD* cardiovascular disease

A CFA of the CESD-8 items demonstrated that our two-factor model distinguishing between somatic and cognitive-affective components fit the data better than the one-factor solution and had good discriminant validity (eFig. 2, SI).

### Cortisol sample

#### TSO model of depressive symptoms

The longitudinal measurement model of depressive symptoms without risk factors had good fit, RMSEA = 0.031, CFI = 0.988, and TLI = 0.984. Based on the variance decomposition described in Prenoveau (2016) [51], on average the overall factor representing the longitudinal persistence of depressive symptoms explained 38% of the model variance, whilst only 24% of variance was occasion-specific (eTable 1), suggesting that symptoms of depression are more stable than episodic in nature.

#### Associations with hair cortisol

**Fig. 3:**
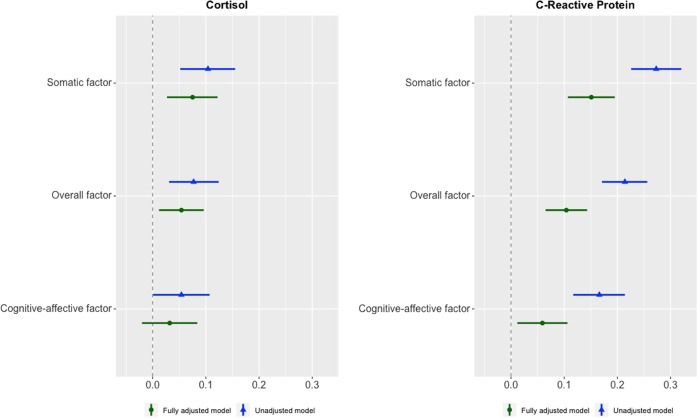
Marginal effects of hair cortisol and C-reactive protein on persistent depressive symptoms: overall, cognitive-affective, and somatic factors. Data source: ELSA, waves 1–8. N: Cortisol = 4761, C-reactive protein = 5784. Unstandardised regression coefficients and confidence intervals. Estimator = WLSMV. Unadjusted model = Model 1 (no covariates). Fully adjusted model = Model 3 (adjusted for demographic, socioeconomic, lifestyle, chronic disease, and medication data)

The marginal effects of cortisol on the overall, cognitive-affective, and somatic factors are shown in Fig. 3 and Table 2. In the unadjusted model (Model 1), higher cortisol levels were significantly associated with greater overall factor scores (*b* = 0.077, 95% CI: 0.030; 0.124). The symptom-specific associations revealed a stronger effect of cortisol on somatic (*b* = 0.104, 95% CI: 0.052; 0.155) than on cognitive-affective symptoms (*b* = 0.054, 95% CI: 0.001; 0.107). Demographic, socioeconomic, and lifestyle characteristics had little impact on these associations (Model 2, Table 2). In contrast, in the fully adjusted model (Model 3, Table 2), these effects reduced considerably when controlling also for chronic disease and medication use. The effect of cortisol on cognitive-affective symptoms was no longer significantly different from zero (*b* = 0.032, 95% CI: −0.020; 0.084). In contrast, the association with the overall (*b* = 0.054, 95% CI: 0.012; 0.096) and somatic (*b* = 0.075, 95% CI: 0.027; 0.122) factors survived after adjustment for all covariates (Model 3, Table 2). Nevertheless, the difference between the effect of cortisol on somatic and cognitive-affective symptoms found in Model 3 was not large enough to reach statistical significance (95% CI: −0.113; 0.027) (Table 2).

**Table 2:** Marginal effects of hair cortisol and C-reactive protein on persistent depressive symptoms: overall, cognitive-affective, and somatic factors

	Overall factor					Cognitive-affective factor					Somatic factor				
	*B*	SE	*p*-value	95% CI	*β*	*B*	SE	*p*-value	95% CI	*β*	*B*	SE	*p*-value	95% CI	*β*
Hair cortisol (*N* = 4761)															
Model 1 (unadjusted)	0.077	0.024	0.001	0.030; 0.124	0.062	0.054	0.027	0.045	0.001; 0.107	0.075	0.104	0.026	<0.001	0.052; 0.155	0.127
Model 2 (partially adjusted)	0.084	0.022	<0.001	0.040; 0.128	0.068	0.060	0.027	0.026	0.007; 0.112	0.068	0.112	0.026	<0.001	0.062; 0.163	0.115
Model 3 (fully adjusted)	0.054	0.021	0.011	0.012; 0.096	0.043	0.032	0.027	0.223	−0.020; 0.084	0.034	0.075	0.024	0.002	0.027; 0.122	0.071
C-reactive protein (*N* = 5784)															
Model 1 (unadjusted)	0.214	0.022	<0.001	0.171; 0.256	0.170	0.166	0.025	<0.001	0.117; 0.214	0.220	0.273	0.024	<0.001	0.226; 0.320	0.325
Model 2 (partially adjusted)	0.145	0.021	<0.001	0.104; 0.186	0.116	0.097	0.024	<0.001	0.049; 0.145	0.112	0.201	0.023	<0.001	0.155; 0.246	0.209
Model 3 (fully adjusted)	0.104	0.020	<0.001	0.065; 0.143	0.082	0.059	0.024	0.015	0.012; 0.106	0.062	0.151	0.023	<0.001	0.107;0.195	0.145
Difference between somatic and cognitive-affective scores															
	Hair cortisol				C-reactive protein										
	Difference	SE	*p-*value	95% CI	Difference	SE	*p-*value	95% CI							
Model 1 (unadjusted)	−0.050	0.037	0.182	−0.123; 0.023	−0.107	0.035	0.002	−0.175; −0.038							
Model 2 (partially adjusted)	−0.052	0.037	0.165	−0.125; 0.021	−0.104	0.033	0.002	−0.169; −0.038							
Model 3 (fully adjusted)	−0.043	0.036	0.234	−0.113; 0.027	−0.092	0.033	0.005	−0.157; −0.026							

Data source: ELSA, waves 1–8 B: regression coefficient. β: standardised regression coefficient. Estimator: WLSMV. Model 1 = unadjusted. Model 2: adjusted for demographic, socioeconomic, lifestyle, and hair (cortisol only) characteristics. Model 3:Model 2+ chronic disease and medication use

*SE* standard error, *CI* confidence interval

### CRP sample

#### TSO model of depressive symptoms

The fit of the longitudinal measurement model of depressive symptoms was good, RMSEA = 0.028, CFI = 0.990, and TLI = 0.986. On average, the overall factor explained 37% of the model variance, whereas 23% of variance was occasion-specific (eTable 2).

#### Associations with CRP

The marginal effects of CRP on the overall, cognitive-affective, and somatic factors are shown in Fig. 3 and Table 2. In the unadjusted model (Model 1), elevated CRP concentration was related to greater overall factor scores (*b* = 0.214, 95% CI: 0.171; 0.256). As for cortisol, the effect of CRP was larger on somatic (*b* = 0.273, 95% CI: 0.226; 0.320) than on cognitive-affective symptoms (*b* = 0.166, 95% CI: 0.117; 0.214) (Table 2). Demographic, socioeconomic, and lifestyle characteristics had a considerable impact on these associations (Model 2, Table 2). The magnitude of the effects of CRP further decreased when controlling also for chronic disease and medication use (Model 3, Table 2). In this fully adjusted model, the marginal effects of CRP on the overall, cognitive-affective, and somatic factors were, respectively: 0.104 (95% CI: 0.065; 0.143), 0.059 (95% CI: 0.012; 0.106), and 0.151 (95% CI: 0.107; 0.195) (Model 3, Table 2). The effect of CRP on somatic symptoms was still substantially larger than that on cognitive-affective symptoms, and such difference was statistically significant (95% CI: −0.157; −0.026) (Table 2).

### Associations with the covariates and sensitivity analyses

The marginal effects of all covariates included in the fully adjusted models can be found in the SI [eTable 3 (cortisol sample), eTable 4 (CRP sample)]. Sensitivity analyses revealed significant differences in socioeconomic, health, and lifestyle characteristics between ELSA participants included in the analysis and those excluded owing to missing biomarker data. Nevertheless, the majority of these effects did not exceed 0.1% [eTable 5 (cortisol sample), eTable 6 (CRP sample)]. Lastly, we tested all models in a subsample of participants with a mean total CESD-8 score of three or more points across waves 1–8 (Cortisol: *N* = 763; CRP: *N* = 801). The pattern of results found in this subsample was similar to that observed in the full analytical sample. Both cortisol and CRP had considerably large effects on the somatic factor, which were robust to adjustment for all covariates. In contrast, their associations with the overall and cognitive-affective factors were much weaker and did not reach statistical significance in most cases, possibly due to the reduced statistical power of these analyses (eTable 7).

## Discussion

This is the first study examining the relationship of hair cortisol and plasma CRP with the persistence and dimensions (i.e., cognitive-affective and somatic) of depressive symptoms across a 14-year period in a large population-based cohort of older adults. As expected, elevated cortisol and CRP levels were associated with persistent depressive symptoms across the study period. Notably, both biomarkers exhibited stronger relationships with somatic than with cognitive-affective symptoms, although such differences were more marked for CRP. The associations of cortisol and CRP with somatic symptoms were independent of relevant demographic, socioeconomic, health, and lifestyle characteristics. In contrast, cortisol was no longer significantly associated with cognitive-affective symptoms after adjustment for all covariates, and the confidence interval for the effect of CRP was close to zero.

Our results of higher CRP and cortisol levels in participants with persistent depressive symptoms corroborate the findings of previous meta-analyses [16, 22, 23]. Several studies have examined HPA-axis function in relation to depression using salivary cortisol levels [52]. The results have generally been positive, suggesting that between 20 and 80% of depressed individuals exhibit some form of HPA-axis hyperactivation [16]. In addition, drugs targeting hormones related to the HPA-axis have been shown to have beneficial effects on depressive symptoms [53]. Likewise, an abundance of studies has indicated that depressed individuals tend to exhibit elevated plasma concentrations of ﻿pro-inflammatory cytokines such as CRP [17]. Furthermore, there is some evidence showing that experimentally induced inflammation can lead to depressed mood [54], whereas anti-inflammatory medication may reduce depressive symptoms [55, 56]. This suggests that HPA-axis hyperactivity and elevated inflammation might be key pathophysiological mechanisms underlying depression, as well as potential mediators of its relationship with stress and physical illness [57]. In line with previous evidence [36], the longitudinal TSO model of depressive symptoms indicated that the proportion of variance explained by time-invariant components was considerably larger than that attributable to time-varying factors. Thus, this demonstrates the value of considering the persistence of depressive symptoms over time. Moreover, since the adverse consequences of stress may take a long time to manifest themselves, persistent depressive symptoms could be a more reliable indicator of the biological embedding of chronic or repeated stress exposure across the life course, particularly amongst older adults [58].

Another key finding of our study is that, as hypothesised, CRP and cortisol had stronger associations with somatic than with cognitive-affective symptoms. For CRP, these results are consistent with previous evidence indicating that higher CRP levels were associated with specific somatic symptoms, but not with cognitive-affective experiences [31–34]. In relation to cortisol, this was the first study to examine the link between HPA-axis function and specific dimensions of depression using hair cortisol. Our results revealed that elevated hair cortisol concentrations were predictive of somatic symptoms, whereas their effect on cognitive-affective symptoms was almost null after adjustment for possible confounding factors. Similar results were also reported by a cohort study of adolescents using salivary cortisol [35]. However, another study did not find clear evidence for a differential relationship of salivary cortisol with the dimensions of depressive symptoms in adolescents [59]. This negative result could be explained by the use of salivary cortisol, which is not a reliable marker of long-term HPA-axis activity [60], or by the young age of the study participants. As expected, the associations of cortisol and CRP with depressive symptoms reduced considerably after adjustment for possible confounding factors. Controlling for the presence of chronic conditions and medication use led to the strongest reduction in the effects of cortisol and CRP on depressive symptoms. This is not surprising given the known bidirectional links of physical illness with depression, neuroendocrine processes, and inflammation [7].

The present findings have important implications and open up new avenues for depression research and treatment. Taken together, they suggest that elevated cortisol and CRP levels could be reliable biomarkers of somatic depressive symptoms, rather than overall depressive symptoms. In addition, they provide further support for the sickness behaviour theory [28, 29], according to which somatic depressive-like symptoms which characterise sickness behaviour are likely to stem from dysregulated inflammatory and neuroendocrine responses. Such somatic changes could in turn influence the development of cognitive-affective symptoms thereby acting as important mediators of the relationship between depression and these biological systems [34]. This possibility is also supported by investigations demonstrating the causal effect of immunotherapy on the development of early-onset somatic depressive symptoms in the majority of cancer patients, while late-onset psychological symptoms occur less frequently [61]. Likewise, immune activation in animals and healthy participants has been shown to lead to typical somatic symptoms of depression [62, 63]. Such results highlight the importance of taking into account specific depressive symptom dimensions in future studies on pathophysiological mechanisms. This could help to advance the search for biomarkers of depression, facilitate more targeted treatments, and inform antidepressant medication selection [64, 65]. To illustrate, since different symptoms may be characterised by distinct biological dysregulations, the efficacy of antidepressants is likely to be affected by the specific symptom profile of patients. In additon, drugs that pharmacologically modify neuroendocrine and inflammatory processes might be particularly effective for individuals with higher levels of somatic symptoms [53, 56].

Our investigation has several strengths. These include, for instance: a large sample size; participants not selected on the basis of mental health issues, and therefore more representative of the general population; robust estimates of depressive symptoms due to repeated measures; and reliable assessment of long-term HPA-axis activity owing to the quantification of cortisol in hair. Notwithstanding this, there also are a number of limitations to consider. First, this study does not provide direct evidence for the possible casual effect of HPA-axis dysfunction and inflammation on depression. Second, we only considered a single biomarker for each biological system, which may not be sufficiently precise to understand the complex role of neuroendocrine and inflammatory processes in depression. Third, sensitivity analyses revealed that ELSA participants with available biomarker measures had better socioeconomic, health, and lifestyle characteristics compared with those who did not participate in the nurse visit or did not have blood/hair samples taken. Last, it is worth noting that there are inconsistencies in the definition of somatic symptoms in the literature [34], as well as in the types of items that characterise this symptom cluster across different depression scales [27]. Therefore, although we employed the most suitable classification of somatic symptoms for the CESD-8 [27, 43], this may not correspond to that used in other studies.

Further work is required to investigate the associations of inflammation and HPA-axis function with the somatic clusters underlying other depression scales, and to elucidate the complex interactions amongst somatic and cognitive-affective symptoms over time [25]. Future studies should also clarify the direction of the associations of depressive symptoms with cortisol and CRP using methods that strengthen causal inference such as genetically informed approaches [66]. Furthermore, it would be worth investigating the relationship of cognitive-affective and somatic symptoms with other biomarkers of HPA-axis function and inflammation, as well as with other biological and environmental risk factors [25].

To conclude, the current study demonstrates that elevated hair cortisol and plasma CRP levels were associated with more persistent depressive symptoms over a 14-year period in a large sample of older adults. Furthermore, their relationship with somatic symptoms was considerably larger than that with cognitive-affective symptoms. These distinct associations reveal the importance of considering symptom-specific effects in future studies on pathophysiological mechanisms. Ultimately, this will have the potential to advance the search for biomarkers of depression and facilitate more targeted treatments.

## Supplementary information

Supplementary Information
