# Supplementary material for: Persistent depressive symptoms, HPA-axis hyperactivity, and inflammation: the role of cognitive-affective and somatic symptoms
Source: Mol Psychiatry. 2019 Aug 21;25(5):1130–40. doi: 10.1038/s41380-019-0501-6 (PMC7192852; doi:10.1038/s41380-019-0501-6)
Supplement: Supplementary file 1 — Supplementary Information [file 41380_2019_501_MOESM1_ESM.doc]

**Supplementary Information**

**eMETHODS**

**Depressive symptoms**

Depressive symptoms were ascertained using the 8-item Centre for Epidemiological Studies-Depression scale (CESD-8)^1^. For each item, participants reported whether or not they had experienced a particular symptom ‘much of the time’ during the previous week. A recent analysis of the CESD-8 items across five cohort studies identified a 3-factor solution represented by negative affect, anhedonia and somatic symptoms^2^. In the present analysis, we grouped item loadings on negative affect and anhedonia into a single cognitive-affective cluster since previous studies have found similar associations of these two dimensions with inflammatory markers^3,4^. This resulted in three items representing a somatic dimension (‘‘everything I did was an effort”, ‘‘sleep was restless”, ‘‘I could not get going”) and five items characterising a cognitive-affective dimension (‘‘enjoyed life”, ‘‘felt depressed”, ‘‘happy”, ‘‘lonely”, ‘‘felt sad”).

**Covariates**

The analyses were adjusted for a number of demographic, socioeconomic, lifestyle, health, and hair characteristics that could affect the relationship of depressive symptoms with cortisol and CRP levels. Demographic variables included sex and age in years. Wealth was used as an indicator of socioeconomic position. This measure was derived from a comprehensive assessment of the participant’s economic resources (e.g. financial, housing and physical wealth) excluding pension wealth, and was categorised into quintiles (1= lowest wealth; 5 = highest wealth). Lifestyle characteristics were represented by smoking status, physical activity, frequency of alcohol use, and body mass index (BMI). Smoking status was a binary variable (yes/no). Physical activity was assessed using data on the frequency of participation in vigorous, moderate and light activities. We dichotomised this variable into two groups: high (vigorous or moderate activity on a weekly basis) and low (no vigorous or moderate activity on a weekly basis) physical activity. For alcohol drinking, participants reported how often they had an alcoholic drink during the past 12 months on a 8-point scale ranging from ”almost every day”(=1) to “not at all in the last 12 months”(=8). BMI was calculated based on measures of height and body weight collected during the nurse visit, and was treated as a continuous variable. Health-related variables included chronic diseases and use of anti-inflammatory, antihypertensive, or antidepressant drugs. The presence of chronic diseases was a binary variable (yes/no) representing lifetime self-reported physician diagnoses of cardiovascular conditions, cancers, chronic lung disease, or diabetes. Anti-inflammatory and antihypertensive medications were grouped into a single binary variable (yes/no). Also the use of antidepressants was treated as a binary measure (yes/no). Lastly, for the cortisol sample, the analyses were adjusted for a number of hair-related characteristics that may influence cortisol levels. These included whether hair was dyed (yes/no), season of hair collection [summer (Jun-Aug), autumn (Sep-Nov), winter (Dec-Feb), spring (Mar-May)], and phase of hair analysis (1=2015; 2=2018).

**Statistical analyses**

Trait-State-Occasion (TSO) models represent an invaluable tool for modelling the longitudinal stability and variability of psychological and behavioural constructs across time. This is achieved by partitioning construct variance into time-varying (i.e. occasion) and time-invariant (i.e. trait) components to examine their associations with other variables. The basic TSO model can be expanded to include method factors corresponding to specific indicators or dimensions underlying a particular construct^5^. The complete specification model used for the analysis as described in Newsom (2015)^6^ is shown in eFigure 1. The observed cognitive-affective and somatic symptom scores were treated as ordinal variables. Consequently, the models were fitted using the robust weighted least squares estimator (WLSMV) in Mplus, which has been designed specifically for modelling categorical variables. The WLSMV estimator handles missing data by estimating parameters and standard errors directly from the available data under the MARX assumption (i.e. missing at random with respect to the covariate variables)^7^. The main drivers of attrition in ELSA are age and socioeconomic position^8^. Thus, since these variables were included in the TSO models, the MARX condition is likely to be satisfied. First, we fitted an unconditional TSO model without risk factors. Second, we fitted a univariate TSO model testing for the unadjusted effects of cortisol and CRP on the three latent factors representing overall (i.e. persistent), cognitive-affective, and somatic symptoms (Model 1). Third, we fitted a multivariate TSO model assessing the effects of cortisol and CRP on the latent factors with adjustment for demographic, socioeconomic, lifestyle, and hair characteristics (Model 2). Lastly, we performed a fully adjusted TSO model also controlling for the presence of chronic diseases and medication use (Model 3).

**Model fit**

The adequacy of the TSO and confirmatory factor analysis (CFA) models was evaluated using Root Mean Square Error of Approximation (RMSEA), Comparative Fit Index (CFI), and Tucker Lewis Index (TLI). RMSEA is a measure of absolute fit. Acceptable model fit is indicated by RMSEA values less than 0.06. CFI and TLI are comparative fit indices which should be greater than 0.90^9^. Additionally, adequate discriminant validity of the two-factor CFA model was determined by a correlation between the two latent factors of less than 0.85^10^.

**eFigure 1. Trait-State-Occasion (TSO) model of depressive symptoms (full specification model).**


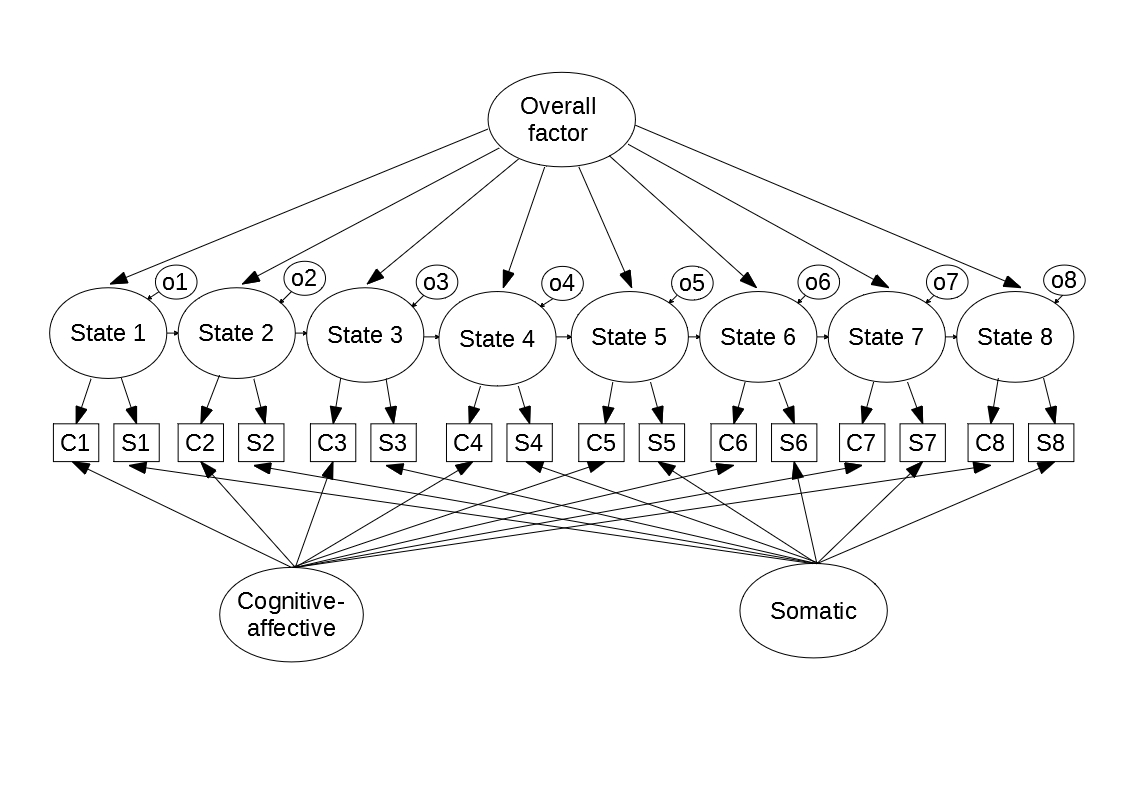


**Legend.** O = Occasion. S = somatic score. C = cognitive-affective score. The TSO model includes state factors for each time point. These are used as second-order indicators of the overall (i.e. trait) factor. The occasion factors represent residual variance in the state factors once the trait variance is removed. The cognitive-affective and somatic (i.e. method) factors correspond to the variance specific to each dimension of depressive symptoms.

**eFigure 2. Confirmatory factor analysis (CFA) of the CESD-8: One-factor versus two-factor solution**

**
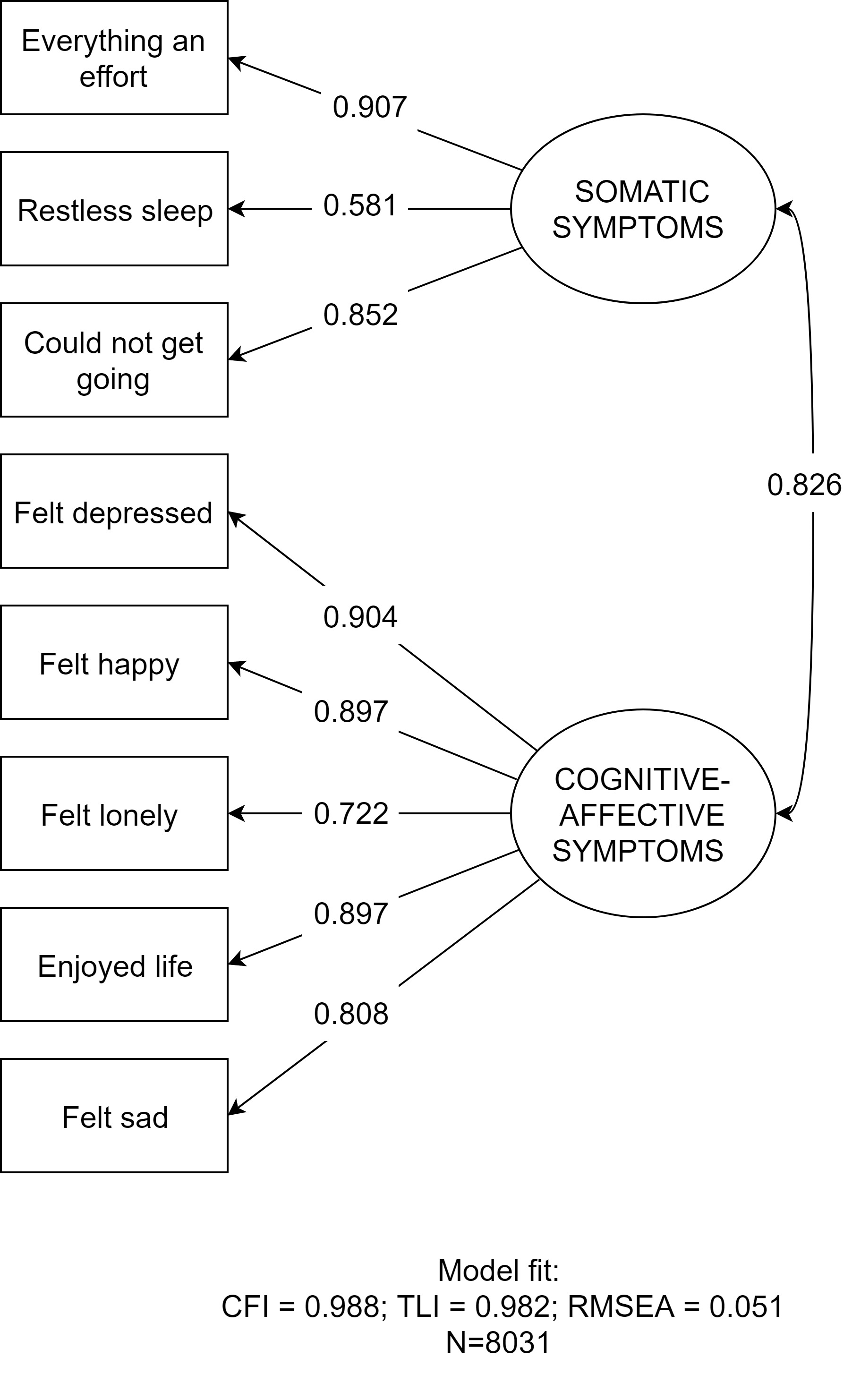

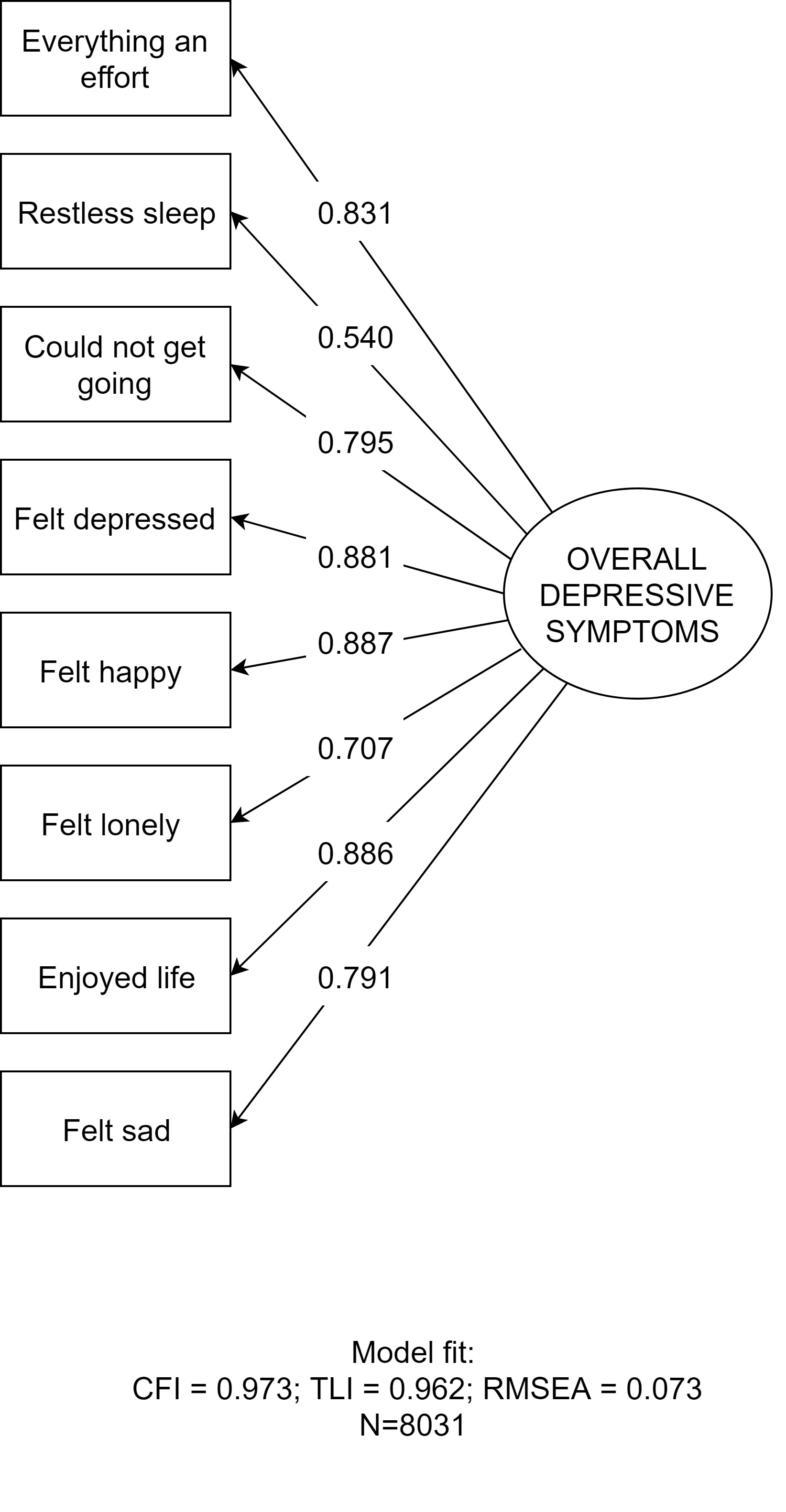
**

**Legend.** The CFA of the CESD-8 items was performed using all ELSA participants in wave 6 with at least one valid response on the CESD-8 scale (N=8031). Estimator: WLSMV.

**References**

1 Radloff LS. The CES-D Scale. *Appl Psychol Meas* 1977; **1**: 385–401.

2 Carleton RN, Thibodeau MA, Teale MJN, Welch PG, Abrams MP, Robinson T *et al.* The Center for Epidemiologic Studies Depression Scale: A Review with a Theoretical and Empirical Examination of Item Content and Factor Structure. *PLoS One* 2013; **8**: e58067.

3 White J, Kivimäki M, Jokela M, Batty GD. Association of inflammation with specific symptoms of depression in a general population of older people: The English Longitudinal Study of Ageing. *Brain Behav Immun* 2017; **61**: 27–30.

4 Duivis HE, Vogelzangs N, Kupper N, de Jonge P, Penninx BWJH. Differential association of somatic and cognitive symptoms of depression and anxiety with inflammation: Findings from the Netherlands Study of Depression and Anxiety (NESDA). *Psychoneuroendocrinology* 2013; **38**: 1573–1585.

5 Prenoveau JM. Specifying and Interpreting Latent State–Trait Models With Autoregression: An Illustration. *Struct Equ Model* 2016; **23**: 731–749.

6 Newsom JT. *Longitudinal Structural Equation Modeling*. Taylor & Francis: New York, 2015.

7 Asparouhov T, Muthén B. Weighted Least Squares Estimation with Missing Data. 2010http://www.statmodel.com/download/GstrucMissingRevision.pdf (accessed 10 Jan2019).

8 Steptoe A, Breeze E, Banks J, Nazroo J. Cohort Profile: The English Longitudinal Study of Ageing. *Int J Epidemiol* 2013; **42**: 1640–1648.

9 Byrne BM. *Structural equation modeling with Mplus: Basic concepts, applications, and programming.* Routledge/Taylor & Francis Group: New York, US, 2012.

10 Kenny DA. Multiple Latent Variable Models: Confirmatory Factor Analysis. http://davidakenny.net/cm/mfactor.htm (accessed 22 May2019).

| **eTable 1. Trait-State-Occasion model parameters of the cortisol sample.** | | | | | | | | |
| --- | --- | --- | --- | --- | --- | --- | --- | --- |
|  | **Total variance** | **R^2^** | | **State Variance** | | **R-squared decomposition** | | |
|  | **R^2^** | **State** | **Method** | **Trait** | **Occasion** | **Trait** | **Occasion** | **Method** |
| *Wave 1* | | | | | |  |  |  |
| Cognitive-affective score | 0.813 | 0.591 | 0.221 | 0.473 | 0.527 | 28.0 | 31.2 | 22.1 |
| Somatic score | 0.795 | 0.680 | 0.104 |  |  | 32.2 | 35.8 | 10.4 |
| *Wave 2* | | | | | |  |  |  |
| Cognitive-affective score | 0.610 | 0.610 | * | 0.956 | 0.044 | 58.3 | 2.7 | * |
| Somatic score | 0.622 | 0.623 | * |  |  | 59.6 | 2.7 | * |
| *Wave 3* | | | | | |  |  |  |
| Cognitive-affective score | 0.752 | 0.621 | 0.131 | 0.596 | 0.404 | 37.0 | 25.1 | 13.1 |
| Somatic score | 0.755 | 0.646 | 0.098 |  |  | 38.5 | 26.1 | 09.8 |
| *Wave 4* |  |  |  |  |  |  |  |  |
| Cognitive-affective score | 0.727 | 0.594 | 0.132 | 0.687 | 0.313 | 40.8 | 18.6 | 13.2 |
| Somatic score | 0.762 | 0.544 | 0.203 |  |  | 37.4 | 17.0 | 20.3 |
| *Wave 5* |  |  |  |  |  |  |  |  |
| Cognitive-affective score | 0.753 | 0.599 | 0.154 | 0.615 | 0.385 | 36.8 | 23.1 | 15.4 |
| Somatic score | 0.759 | 0.616 | 0.131 |  |  | 37.9 | 23.7 | 13.1 |
| *Wave 6* |  |  |  |  |  |  |  |  |
| Cognitive-affective score | 0.758 | 0.598 | 0.159 | 0.604 | 0.396 | 36.1 | 23.7 | 15.9 |
| Somatic score | 0.775 | 0.585 | 0.177 |  |  | 35.3 | 23.2 | 17.7 |
| *Wave 7* |  |  |  |  |  |  |  |  |
| Cognitive-affective score | 0.806 | 0.865 | 0.176 | 0.460 | 0.540 | 39.8 | 46.7 | 17.6 |
| Somatic score | 0.825 | 0.599 | 0.213 |  |  | 27.6 | 32.3 | 21.3 |
| *Wave 8* |  |  |  |  |  |  |  |  |
| Cognitive-affective score | 0.771 | 0.624 | 0.147 | 0.548 | 0.452 | 34.2 | 28.2 | 14.7 |
| Somatic score | 0.806 | 0.558 | 0.234 |  |  | 30.6 | 25.2 | 23.4 |
| Average |  |  |  |  |  | 38% | 24% | 16% |
| **Note.** Data source: ELSA, waves 1-8. N = 4761. Trait = time-invariant variance. Occasion = time-varying variance. Method = symptom-specific variance. ^*^Wave 2 scores were not included in the estimation of the method factors to maintain a positive definite latent variable covariance matrix. | | | | | | | | |

| **eTable 2. Trait-State-Occasion model parameters of the CRP sample.** | | | | | | | | |
| --- | --- | --- | --- | --- | --- | --- | --- | --- |
|  | **Total variance** | **R^2^** | | **State Variance** | | **R-squared decomposition** | | |
|  | **R^2^** | **State** | **Method** | **Trait** | **Occasion** | **Trait** | **Occasion** | **Method** |
| *Wave 1* | | | | | |  |  |  |
| Cognitive-affective score | 0.790 | 0.626 | 0.164 | 0.503 | 0.497 | 31.5 | 31.1 | 16.4 |
| Somatic score | 0.790 | 0.664 | 0.118 |  |  | 33.4 | 33.0 | 11.8 |
| *Wave 2* | | | | | |  |  |  |
| Cognitive-affective score | 0.625 | 0.626 | * | 0.897 | 0.103 | 56.2 | 6.4 | * |
| Somatic score | 0.639 | 0.638 | * |  |  | 57.2 | 6.6 | * |
| *Wave 3* | | | | | |  |  |  |
| Cognitive-affective score | 0.750 | 0.628 | 0.120 | 0.593 | 0.407 | 37.2 | 25.6 | 12.0 |
| Somatic score | 0.761 | 0.640 | 0.114 |  |  | 38.0 | 26.0 | 11.4 |
| *Wave 4* |  |  |  |  |  |  |  |  |
| Cognitive-affective score | 0.735 | 0.588 | 0.146 | 0.672 | 0.328 | 39.5 | 19.3 | 14.6 |
| Somatic score | 0.759 | 0.569 | 0.181 |  |  | 38.2 | 18.7 | 18.1 |
| *Wave 5* |  |  |  |  |  |  |  |  |
| Cognitive-affective score | 0.768 | 0.564 | 0.204 | 0.615 | 0.385 | 34.7 | 21.7 | 20.4 |
| Somatic score | 0.767 | 0.600 | 0.157 |  |  | 36.9 | 23.1 | 15.7 |
| *Wave 6* |  |  |  |  |  |  |  |  |
| Cognitive-affective score | 0.764 | 0.599 | 0.165 | 0.590 | 0.410 | 35.3 | 24.6 | 16.5 |
| Somatic score | 0.783 | 0.584 | 0.189 |  |  | 34.5 | 23.9 | 18.9 |
| *Wave 7* |  |  |  |  |  |  |  |  |
| Cognitive-affective score | 0.793 | 0.624 | 0.170 | 0.496 | 0.504 | 30.9 | 31.4 | 17.0 |
| Somatic score | 0.813 | 0.601 | 0.203 |  |  | 29.8 | 30.3 | 20.3 |
| *Wave 8* |  |  |  |  |  |  |  |  |
| Cognitive-affective score | 0.777 | 0.618 | 0.160 | 0.539 | 0.461 | 33.3 | 28.5 | 16.0 |
| Somatic score | 0.811 | 0.557 | 0.244 |  |  | 30.0 | 25.7 | 24.4 |
| Average |  |  |  |  |  | 37% | 23% | 15% |
| **Note:** Data source: ELSA, waves 1-8. N = 5784. Trait = time-invariant variance. Occasion = time-varying variance. Method = symptom-specific variance. ^*^Wave 2 scores were not included in the estimation of the method factors to maintain a positive definite latent variable covariance matrix. | | | | | | | | |

| **eTable 3. Marginal effects of cortisol and the covariates on persistent depressive symptoms: Overall, cognitive-affective, and somatic factors (Model 3 – fully adjusted).** | | | | | | | | | | | | | | | | |
| --- | --- | --- | --- | --- | --- | --- | --- | --- | --- | --- | --- | --- | --- | --- | --- | --- |
|  |  | **Overall factor** | | | | | **Cognitive-affective factor** | | | | | **Somatic factor** | | | | |
| ***Predictors*** |  | *B* | *SE* | *p-value* | *95% CI* | *β* | *B* | *SE* | *p-value* | *95% CI* | *β* | *B* | *SE* | *p-value* | *95% CI* | *β* |
| **Hair Cortisol (log)** |  | 0.054 | 0.021 | 0.011 | 0.012;  0.096 | 0.043 | 0.032 | 0.027 | 0.223 | -0.020;  0.084 | 0.034 | 0.075 | 0.024 | 0.002 | 0.027;  0.122 | 0.071 |
| **Sex (ref: men)** |  | 0.571 | 0.048 | <.001 | 0.478;  0.665 | 0.215 | 0.456 | 0.059 | <.001 | 0.340;  0.572 | 0.225 | 0.687 | 0.055 | <.001 | 0.580;  0.795 | 0.305 |
|  |  |  |  |  |  |  |  |  |  |  |  |  |  |  |  |  |
| **Age** |  | -0.004 | 0.023 | 0.865 | -0.050;  0.042 | -0.003 | 0.054 | 0.028 | 0.054 | -0.001;  0.108 | 0.056 | -0.031 | 0.027 | 0.250 | -0.083;  0.022 | -0.029 |
| **Wealth (quintiles)** |  | -0.150 | 0.022 | <.001 | -0.194;  -0.107 | -0.159 | -0.082 | 0.024 | 0.001 | -0.129;  -0.036 | -0.114 | -0.091 | 0.023 | <.001 | -0.135;  -0.047 | -0.114 |
| **Smoker (ref: no)** |  | 0.510 | 0.070 | <.001 | 0.373;  0.647 | 0.127 | 0.546 | 0.081 | <.001 | 0.387;  0.705 | 0.179 | 0.526 | 0.079 | <.001 | 0.372;  0.681 | 0.155 |
| **Physical activity (ref: low)** |  | -0.393 | 0.045 | <.001 | -0.481;  -0.305 | -0.154 | -0.336 | 0.054 | <.001 | -0.442;  -0.231 | -0.173 | -0.504 | 0.052 | <.001 | -0.605;  -0.403 | -0.233 |
| **Alcohol use (frequency)** |  | 0.035 | 0.014 | 0.010 | 0.008;  0.062 | 0.060 | 0.021 | 0.014 | 0.134 | -0.006;  0.048 | 0.047 | 0.021 | 0.014 | 0.125 | -0.006;  0.047 | 0.042 |
| **Body mass index (BMI)** |  | 0.040 | 0.030 | 0.187 | -0.019;  0.098 | 0.031 | 0.009 | 0.031 | 0.778 | -0.053;  0.070 | 0.009 | 0.098 | 0.029 | 0.001 | 0.041;  0.154 | 0.089 |
| **Phase of hair analysis** |  | 0.168 | 0.044 | <.001 | 0.082;  0.254 | 0.067 | 0.185 | 0.053 | <.001 | 0.081;  0.289 | 0.097 | 0.155 | 0.050 | 0.002 | 0.057;  0.253 | 0.073 |
| **Any chronic disease (ref: no)** |  | 0.453 | 0.046 | <.001 | 0.363;  0.543 | 0.173 | 0.383 | 0.057 | <.001 | 0.272;  0.494 | 0.192 | 0.573 | 0.053 | <.001 | 0.469;  0.677 | 0.258 |
| **Anti-inflammatory medication (ref: no)** |  | 0.140 | 0.045 | 0.002 | 0.053;  0.228 | 0.056 | -0.015 | 0.054 | 0.783 | -0.121;  0.091 | -0.008 | 0.277 | 0.051 | <.001 | 0.177;  0.377 | 0.130 |
| **Antidepressants**  **(ref: no)** |  | 0.937 | 0.065 | <.001 | 0.810;  1.064 | 0.242 | 0.975 | 0.078 | <.001 | 0.822;  1.128 | 0.330 | 1.004 | 0.074 | <.001 | 0.859;  1.149 | 0.306 |
| **Hair dyed (ref: no)** |  | 0.059 | 0.069 | 0.395 | -0.077;  0.195 | 0.018 | -0.118 | 0.073 | 0.104 | -0.260;  0.024 | -0.048 | -0.038 | 0.070 | 0.584 | -0.175;  0.099 | -0.014 |
| **Season of hair sample collection** | Spring | -0.107 | 0.095 | 0.262 | -0.293;  0.080 | -0.022 | -0.074 | 0.110 | 0.499 | -0.290;  0.141 | -0.020 | -0.089 | 0.109 | 0.414 | -0.304;  0.125 | -0.022 |
| **(ref: summer)** | Autumn | -0.042 | 0.055 | 0.445 | -0.149;  0.065 | -0.017 | -0.041 | 0.067 | 0.537 | -0.172;  0.090 | -0.021 | -0.051 | 0.062 | 0.412 | -0.172;  0.070 | -0.024 |
|  | Winter | 0.021 | 0.060 | 0.729 | -0.097;  0.138 | 0.007 | 0.085 | 0.073 | 0.245 | -0.058;  0.227 | 0.039 | -0.036 | 0.068 | 0.600 | -0.168;  0.097 | -0.015 |
| **Note**. Data source: ELSA, waves 1-8. N = 4761. B = regression coefficient. SE = standard error. β = standardised regression coefficient. CI = confidence interval. Estimator: WLSMV. | | | | | | | | | | | | | | | | |

| **eTable 4. Marginal effects of CRP and the covariates on persistent depressive symptoms: Overall, cognitive-affective, and somatic factors (Model 3 – fully adjusted).** | | | | | | | | | | | | | | | | | |
| --- | --- | --- | --- | --- | --- | --- | --- | --- | --- | --- | --- | --- | --- | --- | --- | --- | --- |
|  | **Overall factor** | | | | | **Cognitive-affective factor** | | | | | | | **Somatic factor** | | | | |
| ***Predictors*** | *B* | *SE* | *p-value* | *95% CI* | *β* | | *B* | *SE* | *p-value* | *95% CI* | *β* | *B* | | *SE* | *p-value* | *95% CI* | *β* |
| **CRP (log)** | 0.104 | 0.020 | <.001 | 0.065;  0.143 | 0.082 | | 0.059 | 0.024 | 0.015 | 0.012;  0.106 | 0.062 | 0.151 | | 0.023 | <.001 | 0.107;  0.195 | 0.145 |
| **Sex (ref: men)** | 0.520 | 0.040 | <.001 | 0.442;  0.599 | 0.204 | | 0.447 | 0.049 | <.001 | 0.352;  0.542 | 0.236 | 0.586 | | 0.046 | <.001 | 0.497;  0.676 | 0.280 |
| **Age** | 0.011 | 0.022 | 0.627 | -0.032;  0.054 | 0.008 | | 0.069 | 0.026 | 0.007 | 0.019;  0.120 | 0.070 | -0.018 | | 0.025 | 0.475 | -0.066;  0.031 | -0.016 |
| **Wealth (quintiles)** | -0.168 | 0.023 | <.001 | -0.214;  -0.122 | -0.175 | | -0.069 | 0.023 | 0.002 | -0.113;  -0.024 | -0.095 | -0.068 | | 0.022 | 0.002 | -0.111;  -0.024 | -0.086 |
| **Smoker (ref: no)** | 0.517 | 0.065 | <.001 | 0.391;  0.644 | 0.128 | | 0.590 | 0.075 | <.001 | 0.442;  0.738 | 0.197 | 0.498 | | 0.071 | <.001 | 0.359;  0.638 | 0.150 |
| **Physical activity**  **(ref: low)** | -0.397 | 0.041 | <.001 | -0.476;  -0.317 | -0.155 | | -0.374 | 0.049 | <.001 | -0.469;  -0.278 | -0.197 | -0.462 | | 0.046 | <.001 | -0.552;  -0.371 | -0.220 |
| **Alcohol use (frequency)** | 0.021 | 0.014 | 0.138 | -0.007;  0.049 | 0.035 | | 0.023 | 0.014 | 0.084 | -0.003;  0.050 | 0.052 | 0.037 | | 0.013 | 0.006 | 0.011;  0.063 | 0.074 |
| **Body Mass index (BMI)** | 0.059 | 0.034 | 0.085 | -0.008;  0.126 | 0.039 | | -0.013 | 0.032 | 0.696 | -0.076;  0.051 | -0.011 | 0.073 | | 0.031 | 0.019 | 0.012;  0.133 | 0.058 |
| **Any chronic disease (ref: no)** | 0.408 | 0.043 | <.001 | 0.323;  0.493 | 0.149 | | 0.324 | 0.052 | <.001 | 0.222;  0.426 | 0.159 | 0.495 | | 0.049 | <.001 | 0.398;  0.592 | 0.219 |
| **Anti-inflammatory medication (ref: no)** | 0.119 | 0.041 | 0.004 | 0.039;  0.199 | 0.047 | | -0.041 | 0.049 | 0.401 | -0.137;  0.055 | -0.022 | 0.242 | | 0.046 | <.001 | 0.152;  0.333 | 0.115 |
| **Antidepressants (ref: no)** | 0.994 | 0.061 | <.001 | 0.874;  1.115 | 0.241 | | 1.082 | 0.074 | <.001 | 0.937;  1.226 | 0.351 | 1.046 | | 0.070 | <.001 | 0.909;  1.184 | 0.308 |
| **Note.** Data source: ELSA, waves 1-8. N = 5784. B = regression coefficient. SE = standard error. β = standardised regression coefficient. CI = confidence interval. Estimator: WLSMV. | | | | | | | | | | | | | | | | | |

| **eTable 5. Characteristics of participants included in the cortisol sample versus excluded participants (wave 6).** | | | | | | | | |
| --- | --- | --- | --- | --- | --- | --- | --- | --- |
|  | Excluded | | | Included | | | Group comparisons | |
|  | **N** | **Mean (%)** | **SD** | **N** | **Mean (%)** | **SD** | ***p-value*^*^** | ***r*^*^** |
| **Cognitive-affective score** | 5139 | 0.65 | 1.23 | 4713 | 0.61 | 1.19 | 0.223 | -0.020 |
| **Somatic score** | 5164 | 0.74 | 0.97 | 4743 | 0.71 | 0.94 | 0.105 | -0.005 |
| **Sex (ref: men)** | 5840 | 0.46 | 0.50 | 4761 | 0.67 | 0.47 | <.001 | 0.213 |
| **Age** | 5840 | 65.13 | 10.22 | 4761 | 67.09 | 9.00 | <.001 | 0.103 |
| **Wealth (quintiles)** | 5683 | 2.94 | 1.42 | 4679 | 3.08 | 1.40 | <.001 | 0.050 |
| **Current smoker (ref: no)** | 5835 | 0.14 | 0.34 | 4761 | 0.11 | 0.31 | <.001 | -0.040 |
| **Physical activity (ref: low)** | 5840 | 0.40 | 0.49 | 4761 | 0.40 | 0.49 | 0.529 | -0.006 |
| **Alcohol use (frequency)** | 4465 | 4.28 | 2.22 | 4365 | 4.44 | 2.23 | <.001 | 0.038 |
| **Body Mass Index (BMI)** | 3139 | 28.33 | 5.06 | 4554 | 28.27 | 5.40 | 0.624 | -0.016 |
| **Anti-inflammatory/**  **hypertensive medication**  **(ref: no)** | 3293 | 0.45 | 0.50 | 4761 | 0.45 | 0.50 | 0.922 | -0.001 |
| **Antidepressants**  **(ref: no)** | 3293 | 0.10 | 0.31 | 4761 | 0.12 | 0.32 | 0.064 | 0.021 |
| **Any chronic disease**  **(ref: no)** | 5840 | 0.36 | 0.48 | 4761 | 0.35 | 0.48 | 0.540 | -0.006 |
| **Note. ^*^***p-value* estimates from significance tests, including t-tests (continuous variables) or chi-square tests (binary variables). *r* coefficients obtained from Spearman’s Rho correlation tests. Means of binary variables are expressed as proportions. | | | | | | | | |

| **eTable 6. Characteristics of participants included in the CRP sample versus excluded participants (wave 6).** | | | | | | | | |
| --- | --- | --- | --- | --- | --- | --- | --- | --- |
|  | Excluded | | | Included | | | Group comparisons | |
|  | **N** | **Mean (%)** | **SD** | **N** | **Mean (%)** | **SD** | ***p-value*^*^** | ***r*^*^** |
| **Cognitive-affective score** | 4110 | 0.71 | 1.29 | 5742 | 0.57 | 1.15 | <.001 | -0.055 |
| **Somatic score** | 4140 | 0.81 | 1.01 | 5767 | 0.67 | 0.91 | <.001 | -0.066 |
| **Sex (ref: men)** | 4817 | 0.56 | 0.50 | 5784 | 0.55 | 0.50 | 0.253 | -0.011 |
| **Age** | 4667 | 65.81 | 10.74 | 5727 | 66.18 | 8.83 | 0.058 | 0.022 |
| **Wealth (quintiles)** | 4677 | 2.87 | 1.43 | 5685 | 3.11 | 1.39 | <.001 | 0.082 |
| **Current smoker (ref: no)** | 4812 | 0.14 | 0.35 | 5784 | 1.11 | 0.31 | <.001 | -0.043 |
| **Physical activity (ref: low)** | 4817 | 0.36 | 0.48 | 5784 | 1.44 | 0.50 | <.001 | 0.084 |
| **Alcohol use (frequency)** | 3514 | 4.51 | 2.30 | 5316 | 4.26 | 2.17 | <.001 | -0.052 |
| **Body Mass Index (BMI)** | 2078 | 29.13 | 5.94 | 5615 | 27.98 | 4.95 | <.001 | -0.084 |
| **Anti-inflammatory/**  **hypertensive medication**  **(ref: no)** | 2270 | 0.51 | 0.50 | 5784 | 0.43 | 0.49 | <.001 | -0.077 |
| **Antidepressants**  **(ref: no)** | 2270 | 0.13 | 0.34 | 5784 | 0.11 | 0.31 | 0.001 | -0.036 |
| **Any chronic disease**  **(ref: no)** | 4817 | 0.41 | 0.49 | 5784 | 0.31 | 0.46 | <.001 | -0.104 |
| **Note. ^*^***p-value* estimates from significance tests, including t-tests (continuous variables) or chi-square tests (categorical variables). *r* coefficients obtained from Spearman’s Rho correlation tests. Means of binary variables are expressed as proportions. | | | | | | | | |

| **eTable 7. Marginal effects of hair cortisol and c-reactive protein on persistent depressive symptoms (i.e. overall, cognitive-affective, and somatic factors) in the sample with a mean total CESD-8 score ≥ 3 across waves 1-8.** | | | | | | | | | | | | | | | | |
| --- | --- | --- | --- | --- | --- | --- | --- | --- | --- | --- | --- | --- | --- | --- | --- | --- |
|  | **Overall factor** | | | | | | **Cognitive-affective factor** | | | | | **Somatic factor** | | | | |
|  | *B* | *SE* | *p-value* | | *β* |  | *B* | *SE* | *p-value* | *β* |  | *B* | *SE* | *p-value* | *β* |  |
| **Hair Cortisol (N = 763)** | | | | | | | | | | | | | | | | |
| **Model 1**  (Unadjusted) | 0.052 | 0.027 | 0.060 | | 0.143 |  | 0.003 | 0.038 | 0.945 | 0.004 |  | 0.108 | 0.034 | <0.001 | 0.161 |  |
| **Model 2**  (Partially adjusted) | 0.043 | 0.027 | 0.118 | | 0.104 |  | -0.011 | 0.039 | 0.784 | -0.015 |  | 0.097 | 0.034 | 0.004 | 0.139 |  |
| **Model 3**  (Fully adjusted) | 0.030 | 0.027 | 0.274 | | 0.070 |  | -0.020 | 0.041 | 0.629 | -0.027 |  | 0.079 | 0.033 | 0.018 | 0.113 |  |
| **C-reactive protein (N = 801)** | | | | | | | | | | | | | | | | |
| **Model 1**  (Unadjusted) | 0.077 | 0.030 | 0.010 | 0.212 | |  | -0.011 | 0.043 | 0.790 | -0.014 |  | 0.169 | 0.036 | <0.001 | 0.238 |  |
| **Model 2**  (Partially adjusted) | 0.075 | 0.031 | 0.015 | 0.189 | |  | -0.023 | 0.043 | 0.587 | -0.027 |  | 0.166 | 0.037 | <0.001 | 0.224 |  |
| **Model 3**  (Fully adjusted) | 0.063 | 0.031 | 0.044 | 0.150 | |  | -0.026 | 0.044 | 0.562 | -0.029 |  | 0.141 | 0.037 | <0.001 | 0.191 |  |
| **Note.** Data source: ELSA, waves 1-8. B = regression coefficient. SE = standard error. β = standardised regression coefficient. CI = confidence interval. Estimator: WLSMV. Model 1 = unadjusted. Model 2 = adjusted for demographic, socioeconomic, lifestyle, and hair (cortisol only) characteristics. Model 3 = Model 2 + chronic disease and medication use. | | | | | | | | | | | | | | | | |
